# Maximizing sinusoidal channels of HZSM-5 for high shape-selectivity to *p*-xylene

**DOI:** 10.1038/s41467-019-12285-4

**Published:** 2019-09-25

**Authors:** Chuanfu Wang, Lei Zhang, Xin Huang, Yufei Zhu, Gang (Kevin) Li, Qinfen Gu, Jingyun Chen, Linge Ma, Xiujie Li, Qihua He, Junbo Xu, Qi Sun, Chuqiao Song, Mi Peng, Junliang Sun, Ding Ma

**Affiliations:** 1 National Institute of Clean-and-Low-Carbon Energy (NICE), 102211 Beijing, China; 2 Beijing National Laboratory for Molecular Sciences, College of Chemistry and Molecular Engineering, Peking University, 100871 Beijing, China; 3 Department of Chemical and Biomolecular Engineering, The University of Melbourne, Melbourne, VIC 3010 Australia; 4 Australian Synchrotron, 800 Blackburn Rd, Clayton, VIC 3168 Australia; 5 Dalian Institute of Chemical Physics, Chinese Academy of Sciences, 116023 Dalian, China; 6 Peking University Health Science Centre, 100191 Beijing, China; 7 Institute of Process Engineering, Chinese Academy of Sciences, 100080 Beijing, China; 8 College of Engineering and BIC-ESAT, Peking University, 100871 Beijing, China

**Keywords:** Catalyst synthesis, Heterogeneous catalysis, Chemical engineering

## Abstract

The shape-selective catalysis enabled by zeolite micropore’s molecular-sized sieving is an efficient way to reduce the cost of chemical separation in the chemical industry. Although well studied since its discovery, HZSM-5′s shape-selective capability has never been fully exploited due to the co-existence of its different-sized straight channels and sinusoidal channels, which makes the shape-selective *p*-xylene production from toluene alkylation with the least *m*-xylene and *o*-xylene continue to be one of the few industrial challenges in the chemical industry. Rather than modifications which promote zeolite shape-selectivity at the cost of stability and reactivity loss, here inverse Al zoned HZSM-5 with sinusoidal channels predominantly opened to their external surfaces is constructed to maximize the shape-selectivity of HZSM-5 sinusoidal channels and reach > 99 % *p*-xylene selectivity, while keeping a very high activity and good stability ( > 220 h) in toluene methylation reactions. The strategy shows good prospects for shape-selective control of molecules with tiny differences in size.

## Abstract (web summary)

Full utilization of ZSM-5 shape-selectivity is restricted by crystal external surface acid sites and co-existence of two different sized channels. Here, the authors synthesize reverse Al zoned ZSM-5 with sinusoidal channel preferentially opened to its inert external surface to achieve 99.3% *p*-xylene selectivity.

## Introduction

Shape-selective catalysis manipulates the interplay between the structure of zeolites (catalyst) and the dimension/configuration of reactants/products/intermediates to control the selectivity towards desired products or the reaction pathways in a catalytic reaction^1–3^. An ideal shape-selective catalyst should have a precise control on the structure and size of its pores^4^, of which the dimension only permits the pass of molecules smaller than the size of the zeolite’s pores, i.e., the molecular sieving effect^5^. However, precise design and synthesis of zeolites with the pore size suitable for specific molecules is extremely challenging, especially when the molecular structures of the intermediates/products involved in a reaction are only slightly different. Frequently, zeolite pore-size fine-tuning by modifiers is necessary to improve shape-selectivity. HZSM-5 is a well-known shape-selective zeolite that showed very high selectivity toward *para*-xylene (*p*-xylene), the most desirable xylene isomer in methanol–toluene alkylation reaction, when its channels were modified by P^6–11^, B^7,8,12^, SiO_2_^13–20^, or Mg^6,8,19,21,22^ to match the kinetic size of *p*-xylene. Although efficient and effective, modified HZSM-5 had to compromise its reactivity with selectivity since the modifiers acted as acid-sites neutralizing reagent and partially blocked the pore to narrow its openings, which may eventually lead to deteriorated diffusivity of reactants, loss of useful active sites, pore volume reduction, and reduction of available surface area^10,23–27^. Thus, in the end, modified HZSM-5 showed lowered specific activity (Supplementary Table 1). Moreover, these modified zeolites were susceptible to modifiers loss and thus showed poor stability in long-run reactions^23,24,26^. These drawbacks are major challenges for the development of practical HZSM-5 catalysts with superior shape-selectivity to *p*-xylene, which aims to get highly purified *p*-xylene with low energy consumption^28^.

Except for modifications to HZSM-5 catalyst, *p*-xylene selectivity can also be enhanced to some extent by maximizing xylene isomers’ diffusion resistance in HZSM-5 pores, for example, large HZSM-5 crystals always show higher selectivity to *p*-xylene with elongated transport length^29–31^. In this regard, HZSM-5′s shape-selective capability can be further exploited by differentiating its channels. HZSM-5 has two groups of intersecting channels with similar opening sizes but different shapes. As the 0.51 × 0.55 nm sinusoidal channels are smaller and more tortuous than the 0.52 × 0.58 nm straight channels, xylene molecules are subjected to lower diffusion resistance when travelling in HZSM-5′s straight channels than in the sinusoidal channels. Therefore, straight channels are less efficient for xylene isomers shape/size sieving and are possibly one of the main reasons for the generation of undesired *m*-xylene and *o*-xylene in the methanol–toluene alkylation reaction. However, the co-existence of the two different pore systems makes the shape-selective capability of HZSM-5 hard to be fully exploited^32^.

To maximize the shape-selective ability of HZSM-5 and to get ultra-high *p*-xylene selectivity in methanol–toluene alkylation reaction, is it possible to design a HZSM-5 catalyst with only effective sinusoidal channels? Here we report the investigation of HZSM-5 with dominantly sinusoidal channel openings over its acid-sites free external surface to enforce xylene’s diffusion, which enabled both good reactivity for toluene conversion and selectivity (more than 99%) towards *p-*xylene in a 220 h methanol–toluene alkylation reaction. Our catalyst design strategy may reduce the energy consumption of *p*-xylene industry in large scale.

## Results

### Zeolites performance

**Fig. 1:**
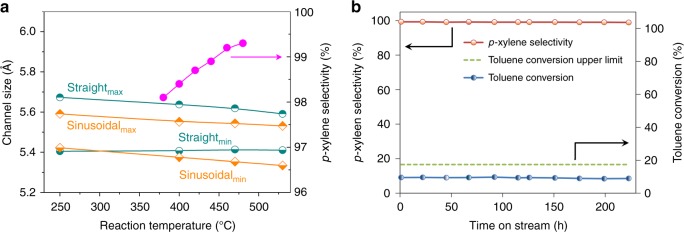
Catalytic performances of HZSM-5_T catalyst in methanol–toluene alkylation reaction. **a** Effects of reaction temperature on ZSM-5 shape-selectivity to *p*-xylene, and on ZSM-5 straight channel and sinusoidal channel sizes, where subscript max and min refers to the longest and shortest diameter of the elliptical cross-section of ZSM-5′s channel respectively, obtained from computed crystallographic data in Supplementary Fig. 3 and Supplementary Table 2. **b** Toluene conversion and *p*-xylene selectivity evolution with time on stream, where the upper limit of toluene conversion marked with dashed green line is 16.6% (470 °C; atmospheric pressure; toluene/methanol (mol/mol) = 6 was co-fed with hydrogen and water; see Methods for details)

Four HZSM-5 samples, with similar overall Si/Al ratios but different morphology/structure, were used in this study. Among them, two coffin-shape HZSM-5 with different particle sizes: HZSM-5_C1 and HZSM-5_C2 (SEM image in Supplementary Fig. 1), were prepared following reported procedures^33,34^. Twin HZSM-5 (labelled as HZSM-5_T) with intergrowth structure was prepared followed a procedure described in the Methods section. A commercial HZSM-5 purchased from NanKai Catalyst Factory (termed as HZSM-5_NK) was used as reference. These catalysts were all evaluated in the methanol–toluene alkylation reaction at 470 °C. Clearly, all these HZSM-5 catalysts have shape-selective effect except HZSM-5_NK, over which only thermodynamic equilibrium *p*-xylene selectivity of 24.1% was obtained. For HZSM-5_C1 and HZSM-5_C2, the selectivity towards *p*-xylene (see Table 1) could not achieve an ultra-high *p*-xylene selectivity required for robust separation/downstream utilization. Much to our surprise, by the HZSM-5_T catalyst, a 99.3% *p-*xylene selectivity was reached, surpassing the three control catalysts. Breen et al.^25,26^ had reported a high *p*-xylene selectivity in toluene alkylation reaction. However, high selectivity could only be obtained over modified HZSM-5 with a low reactant-catalyst contact time by recirculating large volume of hydrogen gas. Instead, for our HZSM-5_T catalyst, the superior selectivity toward *p*-xylene could be reached on a broad range of contact time and toluene-to-methanol ratios, as shown in Supplementary Fig. 2. This has not been reported before and could reduce the operation cost for gas recirculation in industrial applications. Meanwhile, we did notice a slight but continuous increase in *p-*xylene selectivity with increasing reaction temperatures, as shown in Fig. 1a. Considering that thermodynamics equilibrium level of *p-*xylene is not influenced by reaction temperature^35^, this selectivity increase is expected to be a result of ZSM-5 pore shrinkage as shown in Fig. 1a, which suggests the pore narrowing could improve the shape-selectivity of HZSM-5 to *p-*xylene. Significantly, the HZSM-5_T catalyst showed very high specific reactivity compared with the reference catalysts reported in literature (Supplementary Table 1) and extremely stable catalytic performance in a 220-h methanol–toluene alkylation reaction, confirming the superiority of current zeolite HZSM-5_T (Fig. 1b).

**Table 1:** Catalytic performances of HZSM-5 with different aluminium distribution properties to produce *p*-xylene

Catalyst	C_T_, (%)	*p*-xylene sele. (%)	Xylene sele. (%)	Si/Al ratio	
				XRF	XPS
ZSM-5_NK	13.6	24.1	91.2	170	32.6
ZSM-5_C1	10.6	81.9	94.3	135	>1000
ZSM-5_C2	11.7	31.1	79.2	150	64
ZSM-5_T	10.3	99.3	99.0	148	>1000

*C*_*T*_ toluene conversion. See the calculation of toluene conversion and product selectivity in the Methods section.

### Inverse aluminium zoning

**Fig. 2:**
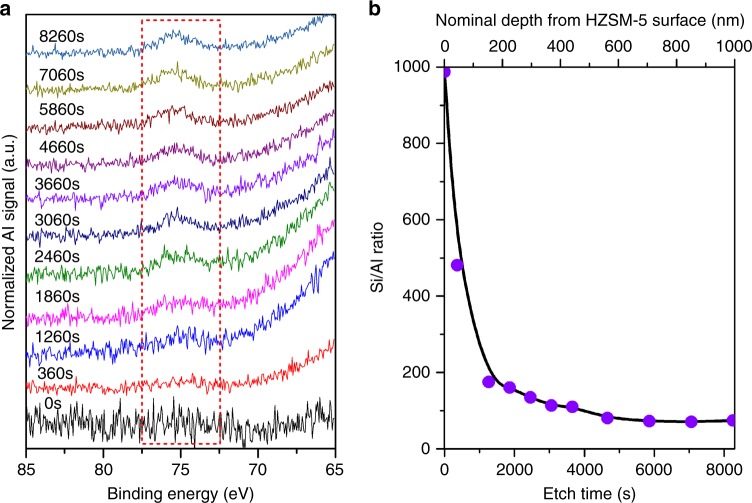
XPS depth profile of HZSM-5_T. **a** Evolution of normalized Al 2p XPS peak (marked by dotted red rectangle) intensity with Ar-ion sputtering time and **b** dependence of the Si/Al ratio on Ar-ion sputtering (etch time, bottom axis) and nominal depth (upper axis) from HZSM-5_T surface (based on Ar-ion sputtering over Ta_2_O_5_ film, of which the thickness was reduced by 0.17 nm every second under the same sputtering conditions)

We were curious why HZSM-5_T could stand out of the zeolites with superior shape-selectivity. The larger zeolite crystals were one of the reasons since size increase always means longer diffusion path. Niwa et al.^31^ has found there was relevance between shape-selectivity and HZSM-5 crystal size, i.e., large HZSM-5 crystals generally showed higher selectivity to *p*-xylene than small ones. Another reason, for the low shape-selectivity of pure HZSM-5, a prevailing view is the occurrence of non-shape-selective reactions over HZSM-5 crystals’ external acid sites^19,31^. A surface treatment, which resulted in chemically inert silica or carbon coating on the external surface, was often applied to passivate its external surface acid sites to increase shape-selectivity^13–19,36–39^. In our study, it was interesting to find that the intergrowth HZSM-5_T actually has an intrinsic inert external surface with aluminium gradient from the surfaces to the core (low to high), as evidenced by the increase of normalized Al signal with extension of etching time when Ar ion was used to remove the surface layer of HZSM-5 (Fig. 2a), as well as the decrease of Si/Al ratios from surface to core illustrated in Fig. 2b. As the number of acid sites is associated with the tetrahedral aluminium species in HZSM-5 (Supplementary Fig. 4), and the dominant aluminium species in HZSM-5_T is tetrahedral aluminium as determined by ^27^Al MAS NMR (Magic-angle Spinning Nuclear Magnetic Resonance) spectroscopy (Supplementary Fig. 5), we believe that HZSM-5_T indeed has acid sites density gradient from surface to core, with very few acid sites on the external surface of the crystals to avoid the non-shape-selective reactions, and most of the acid sites reside in the inner core. It should be noted the acid sites density enhancement in zeolite cores is regardless of the overall Si/Al ratios. When HZSM-5_T samples with a broad range of Si/Al ratios were crushed to expose their internal surface (see SEM image in Supplementary Fig. 6) for XPS (X-ray photoelectron spectroscopy) tests, all these samples showed decreased Si/Al ratios (Supplementary Fig. 7), indicating an increase of acid sites density from surface to core. However, it does not necessarily mean that ZSM-5 crystals with poor acid-sites surface are sufficient to achieve high *p*-xylene selectivity. Of the four zeolites used, coffin-shape HZSM-5_C1 (see SEM image in Supplementary Fig. 1), with similar particle size to that of HZSM-5_T and acid sites density gradient from surface to core, as well as very high surface Si/Al ratio (Supplementary Fig. 8), only showed 81.9% selectivity to *p*-xylene (see Table 1). Meanwhile, the HZSM-5_C2 and HZSM-5_NK catalysts with enriched surface acid sites and lower Si/Al ratio determined by XPS than that determined by XRF (X-ray fluorescence) did exhibit much lower shape-selectivity to *p*-xylene than HZSM-5_C1.

The zoned Al distribution was first discovered by Balloom et al.^40^ and Al zoning seemed to be more common with large ZSM-5 crystals over which Al rich rims were frequently observed. It is sure the Al zoning has close relationship with the recipe of zeolite synthesis and the procedures. Although the exact reasons lead to this reverse Al zoning is not known yet, the large crystal reason can be excluded here since both Balloom et al.^40^ and Groen et al.^41^ reported Al rich ZSM-5 crystal rim with crystal size ranged from 25 to 50 μm. It should be noted although the HSM-5_T samples with different Si/Al ratios all showed reverse Al zoning (Supplementary Fig. 7), the dependence of *p*-xylene selectivity on zeolite overall Si/Al ratios, especially on the external surface Si/Al ratios, was still observed, as shown in Supplementary Table 3. A surface passivation achieved by inert Silicate-1 coating^39^ (SEM image in Supplementary Fig. 9) was still effective to further improve their shape-selectivity to *p*-xylene to some extent, which showed the critical roles of surface acid sites in the shape-selectivity process.

### Sinusoidal channels

**Fig. 3:**
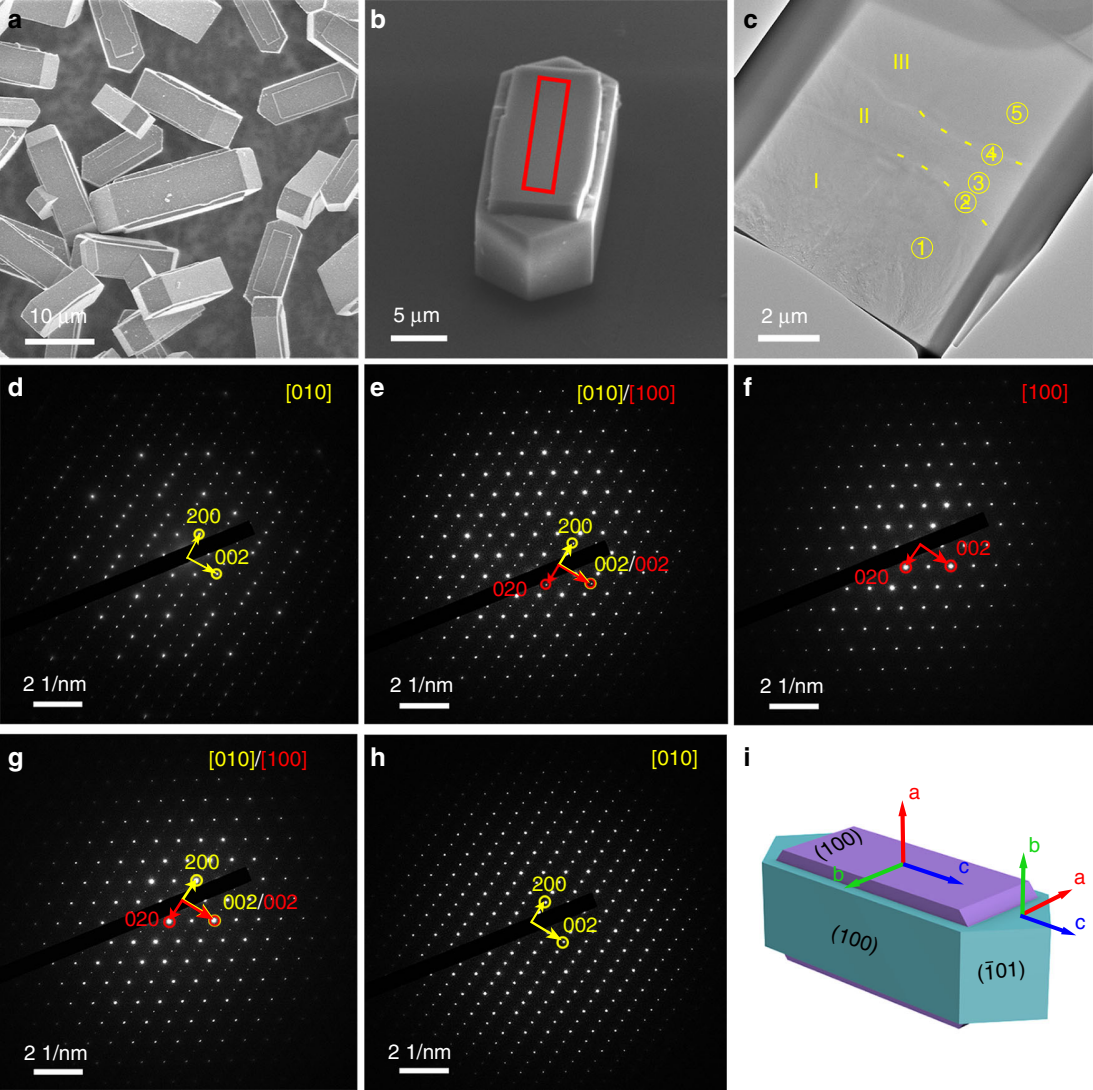
Structural details of HZSM-5_T crystal. **a** SEM images of HZSM-5_T crystals showing a highly intergrowth morphology, with the front facet of each main crystal covered by corresponding intergrowth twin crystal. **b** A thin cross-section cut from the central part of this crystal (marked with a red rectangle) was fabricated by FIB. **c** TEM side view of the lamella taken from **b**. The lamella contains three domains that are separated by two boundaries partially highlighted by yellow dashed lines. The areas from area ① to ⑤ were selected for SAED. **d**–**h** show SAED patterns correspond to areas in **c** from ① to ⑤, respectively. Based on the unit cell parameters and *Pnma* symmetry, the main indexes are marked in yellow and red for different twin domains. **i** The deduced twinning model of HZSM-5 crystals, where the two twin components, marked in blue and purple, differ in their crystallographic orientation by 90° rotation over the common *c*-axis

Since the inert HZSM-5_C1 external surface could restrict the non-shape-selective reactions, i.e., the reactions lead to the formation of *m*-xylene and *o*-xylene, we are eager to know where *m*-xylene and *o*-xylene have come from HZSM-5_C1 and how the morphology of HSM-5_T and HZSM-5_C1 influenced their formation differently. SEM and selected-area electron diffraction (SAED) were used to identify the structure of HZSM-5_T. As shown by the SEM image in Fig. 3a, the HZSM-5_T crystals have a highly intergrowth morphology. For each crystal, most areas of its front facet were covered by an intergrowth twin crystal. In order to get the detailed orientations of different twinning domains, a slice of an intergrowth crystal was chopped out and milled by focused ion beam (FIB) into a lamella (Fig. 3b). As shown in Fig. 3c, the TEM (Transmission Electron Microscopy) image shows a light hourglass shape in the middle area of the lamella (partially indicated by yellow dashed lines). The hourglass shaped area divides the lamellar cross-section into three iso-chemical component domains, namely I, II, and III. Subsequently, five areas in the lamella, two outside ones (①, ⑤), two boundary ones (②, ④), and the one inside the hourglass shaped area (③), were selected to record the SAED patterns (Fig. 3d–h). Areas ① and ⑤ and domains I and III indicate the single crystal feature along the (010) direction (see Fig. 3d–h, respectively), whereas area ③ and domain II illustrate the single crystal feature along the (100) direction (Fig. 3g); the boundary areas ② and ④ depict the patterns along both (100) and (010) directions (Fig. 3f–h). Figure 3i shows the sandwich-like twinning model of HZSM-5_T with orientation of each piece.

Detailed orientation of the channels of this highly intergrowth HZSM-5_T could also be characterized by laser confocal fluorescence microscopy, which was demonstrated clearly by the pioneer works of Weckhuysen et al.^42–44^ and Roeffaers et al.^32,45,46^ They used this technology to study the structure of zeolite and diffusion behaviour of various molecules in zeolite channels. Here, with furfuryl alcohol as the probe molecule, which emits strong fluorescence when catalysed by Brönsted acid sites, the internal structure of the twinned HZSM-5_T can be determined. Layer by layer depth scan profiles along both *b* and *a* axes of two twinned ZSM-5 crystals, which lies on their (100) and (010) facet, respectively, were shown in Supplementary Fig. 10. We observed the edge of each crystal, regardless of the crystal lying with its (010) or (100) facet, are less illuminated. This has been proved by Roeffaers et al.^32^ to be a result of the preferential occupation of oligomers in straight channel when water was used as solvent. The straight channels run parallel to the direction of polarized excitation light and thus the oligomer fluorophores in straight channels showed the strongest fluorescence emission. The existence of the areas without illumination is because that the straight channel runs perpendicular to the polarized excitation light and sinusoidal channels lacks fluorophores when water was used as solvent^32^. The depth scan also confirmed the hourglass shape in the interior of the HZSM-5_T crystal determined by TEM in Fig. 3c.

A schematically model of the intergrowth structure, based on our SAED result and the confocal fluorescence microscopy layer scan (Supplementary Fig. 10), is shown in Supplementary Fig. 11. From this model, it is clearly seen that the intergrowth structure was formed by two independent hemispherical twin crystals embedded in the main crystal. Over the hemispherical twin crystal, there are only no-through sinusoidal channels growing along their *a*-axis, with one end of the channel open to the external surface that can be accessed by molecules, and another end intercepted by the main crystal. The pore openings of the twin crystal straight channels were nearly fully covered by the main crystal and gave very limited access to molecules. Over the main crystal, the same situation existed, i.e., only sinusoidal channels pore openings were retained, and the straight channels were largely blocked by the capped twin crystals from both sides, which forms the hourglass shape observed in Supplementary Fig. 10a and Fig. 3c. As a result of the intergrowth structure, it was determined that, by measuring the dimensions of each single crystal, sinusoidal pore openings were calculated to account for 73% of the total openings on the external surface of the intergrowth HZSM-5_T used in this study. For comparison, over a classic coffin-shape ZSM-5 crystal with the same dimensions, sinusoidal pore openings only accounted for 43% of the total pore openings.

## Discussion

**Fig. 4:**
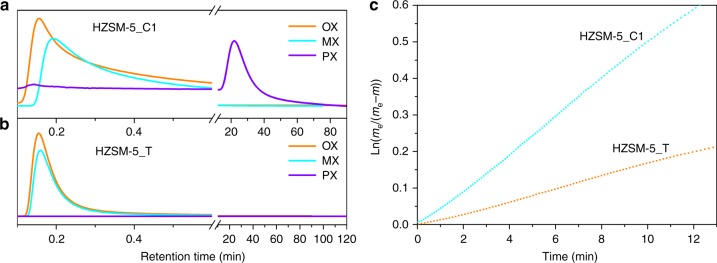
Comparisons of xylene isomers adsorptions over different zeolite catalysts. Pulse chromatography of xylene isomers (OX: *o*-xylene; MX: *m*-xylene; PX: *p*-xylene) passing through columns packed with coffin-shape HZSM-5_C1 (**a**) and **b** twinned HZSM-5_T, at 220 °C. Peak tailing and residence time difference between *m*-xylene and *o*-xylene was observed due to the existence of more straight channels openings and easier entry of *m*-xylene and *o*-xylene into the straight channels of coffin-shape HZSM-5 when compared to twinned HZSM-5. **c** Room temperature *m-*xylene adsorption rate over coffin-shape HZSM-5_C1 and twinned HZSM-5_T showing a lower *m*-xylene adsorption rate when less straight channels were involved in the adsorption process. Data were taken with *m*-xylene equilibrium pressure 1.3 mbar and fitted with a Lagergren adsorption equation^52^, where *m*_e_ and *m* were the quantity of *m*-xylene absorbed at equilibrium and at time *t*, respectively, and the slope of the fitted line was the adsorption rate

It is expected that this surface pore opening difference of intergrowth HZSM-5_T would bring immense influence to the adsorption of xylene isomers, since smaller and tortuous sinusoidal channels are involved. For verification and comparison, adsorption of xylene isomers over intergrowth HZSM-5, and similar-sized single crystal coffin-shaped HZSM-5_C1 (SEM in Supplementary Fig. 1, sinusoidal pore openings accounted for 30% of the total pore openings) with unobstructed straight channels (SAED in Supplementary Fig. 12), was tested by pulsed chromatography and by applying the theory of size exclusion column, i.e., molecules that cannot enter the pore would elute first (the minimum retention time) because they have the least volume to move, while molecules that can enter the pores of HZSM-5 would elute the last (the maximum retention time) due to more volume to travel. Here, rather than the reaction temperature 470 °C, a lower temperature 220 °C was used for the pulsed chromatography experiment since 250 °C is high enough to cause the isomerization reaction of xylene isomers^47,48^. As shown in Fig. 4a, over classical coffin-shape HZSM-5_C1 with unobstructed pore openings and without internal barriers, *o*-xylene flowed out of the HZSM-5 packed column with the least retention, since *o*-xylene molecules have the largest size among the three xylene isomers and are the most difficult to enter either straight pore or sinusoidal pores. Compared to *o*-xylene, *m-*xylene molecule is relatively smaller and thus easier to diffuse into the micropores of ZSM-5, which lead to its higher retention time in the column. *p*-Xylene is the smallest molecule among all the isomers and can be absorbed and desorbed by classic ZSM-5 micropores easily, and thus showed the longest retention time. Meanwhile, the expanding and tailing of *o*-xylene and *m-*xylene peaks also suggested both *o*-xylene and *m-*xylene could still enter into the pores of the coffin-shaped ZSM-5-C1, although with much more difficulty. For comparison, over highly intergrowth HZSM-5_T, as shown in Fig. 4b, although both *m-*xylene and *o*-xylene peaks showed slightly tailing, clearly two isomers flowed out of the column without significant retention time difference, indicating even *m-*xylene molecules were experiencing difficulty to enter the pores of the intergrowth HZSM-5_T. For *p*-xylene, under our experiment conditions, however, could not flow out of the packed column within 2 h. This was considered to be a result of that *p*-xylene molecules were unable to overcome the energy barrier under the test conditions to diffuse out of the sinusoidal channels, due to the twinned structure of HZSM-5_T. For the other two surface aluminium-rich HZSM-5 samples, HZSM-5_NK also showed less capability of preventing the entry of *m*-xylene into its pores, while HZSM-5_C2 seemed to have difficulty to permit the entry of even *p*-xylene, since *p*-xylene flow out of the zeolite column shortly after the injection with a retention time of only 2.17 min, as shown by the pulsed chromatography result in Supplementary Fig. 13. On the other hand, the adsorption distinction between coffin-shape HZSM-5_C1 and HZSM-5_T was also evidenced by their adsorption rate difference to *m-*xylene, the second smallest xylene isomer and supposed to be more sensitive to shape-selectivity change^49^. Coffin-shape HZSM-5_C1 with unobstructed straight channels is more likely to adsorb *m*-xylene than HZSM-5_T, as shown in Fig. 4c, indicating the effectiveness of this intergrowth structure to distinguish xylene isomers. This may explain why even surface inert HZSM-5_C1 or surface passivated coffin-shape HZSM-5 is not enough to achieve high *p*-xylene selectivity, since their straight channels are not small enough to prohibit the diffusion of *m*-xylene^39^. However, even though, we could not exclude the inferior shape-selectivity of HZSM-5_C1 caused by its shorter diffusion path, since HZSM-5_C1 has obvious shorter *b*-axis than that of HZSM-5_T.

Thus, it is concluded that the twinned HZSM-5_T crystals synthesized in this study possessed a decreasing acid site density gradient from the core to the surfaces. Over this acid-in-core HZSM-5_T, xylene molecules have to diffuse through the sinusoidal channels and thus favour the formation of *p*-xylene molecules. The acid sites poor surface prohibited the non-shape-selective reactions that happened on the external surface of zeolite or in the near pore mouth area due to the less availability of reactive centres, which contributed to the high selectivity of *p*-xylene in the final product. Surface with high Si/Al ratio is hydrophobic and thus more resistant to the steam-caused dealumination under the high steam concentration atmosphere reaction conditions^50,51^, which contributes to the excellent stability of our catalysts observed in Fig. 1a. This study provided a new and much more effective strategy for the control of zeolite shape-selectivity, especially for the industrialization of high-performance zeolites for *p*-xylene production without affecting zeolite’s catalytic reactivity.

## Methods

### Zeolite synthesis

HZSM-5_T was prepared by a traditional hydrothermal method. Specifically, NaAlO_2_ (aqueous solution 0.15 mol/L) was first mixed with TPABr (aqueous solution, 1.7 mol/L) to get the turbid sol, followed by sequential addition of silica gel, n-butylamine and deionized water. All these operations were finished at room temperature and under magnetic agitation. The molar ratio of the obtained mixture was 300 SiO_2_:1 Al_2_O_3_:1 Na_2_O:13.5 TPABr:106 n-Butylamine:10160 H_2_O. The mixture was further agitated for 30 min and sealed into a rotating autoclave and heated at 180 °C for 48 h. Then, the sample was cooled down and after filtration, it was calcined in air at 550 °C for 6 h to get the HZSM-5 with highly twinned structure, which we termed as HZSM-5_T. Typically, HZSM-5_T has an Si/Al ratio of 150 and a surface area of 380 m^2^/g.

### Pulsed chromatography test

Pristine powder HZSM-5 was packed in a 100 mm 1/8″ stainless steel tube through vacuum suction to form an adsorption column. The adsorption column was connected to an Agilent 7890B GC equipped a flame ion detector (FID). A G4513A auto-sampler was used to ensure a precise control of the retention time, and the retention time reproducibility of xylene peaks typically fall within a very tight range of ±0.001 min. The GC oven temperature was controlled at 220 °C to ensure the least isomerization during the adsorption test.

### Synchrotron XRD test

HZSM-5-T zeolite powder sample was loaded into 0.7 mm diameter quartz capillary for high temperature in situ powder X-ray diffraction (PXRD) measurement at PD beamline, Australian Synchrotron (ANSTO). Sample was heated up from room temperature to 530 °C with an in-house built hot air blower. The wavelength was calibrated to be 0.7296 Å with a NIST LaB6 standard. The PXRD data were refined with TOPAS 5 software (Bruker) and the result was shown in Supplementary Fig. 3 and Supplementary Table 2.

### Catalytic reaction

Typical reaction conditions are: 0.2 g powdered ZSM-5, atmospheric pressure, 470 °C, toluene/methanol (mol/mol) = 6 mixture was used for higher methanol efficiency (Supplementary Fig. 2) and water was fed to the reactor simultaneously and separately at 0.03 mL/min by two Shimazu HPLC pumps. In all, 55 mL/min hydrogen (controlled by Brooks 5500E mass flow controller) was co-fed to the reactor unless specified. The effluent of the reactor was maintained at 180 °C and introduced into an Agilent 7890B GC equipped with a sampling valve, an FID (flame ionization detector) and an INNOWAX capillary column for online analysis. Methanol conversion was normally 100% and xylene account for 99% of the products. *p*-Xylene selectivity was defined as the proportion of *p*-xylene in total xylene isomers. Toluene conversion was calculated by the following formula:

$${\mathrm{C}}_{\mathrm{T}}\left( \% \right) = \frac{{\mathop {\sum}\limits_{i = 7}^{10} {{\mathrm{C}}_n{\mathrm{H}}_{2n - 6}} }}{{1 + \mathop {\sum}\limits_{i = 7}^{10} {{\mathrm{C}}_n{\mathrm{H}}_{2n - 6}} }} \times 100{\mathrm{\% }},$$

where C_T_ is the conversion of toluene and C_*n*_H_2*n*−6_ (carbon numbers from 7 to 10) is the mole of aromatics detected by GC.

Methanol efficiency (Supplementary Information Fig. 2a) was calculated by the following formula:

$$\eta = \frac{{{\mathrm{C}}_{\mathrm{T}}}}{{{\mathrm{C}}_{{\mathrm{max}}}}} \times 100\%,$$

where *η* is the efficiency of methanol, C_T_ is the conversion of toluene, and C_max_ is the theoretical upper limit of toluene that can be reached with the specific toluene to methanol molar ratio, supposing that the methyl group in methanol was 100% transferred to toluene. For example, with a toluene to methanol molar ratio of 6, the C_max_ is 16.6%.

## Supplementary information

Supplementary Information

Peer Review

Source Data

## Data Availability

The data that support the findings of this study are available from the corresponding author C.W. upon reasonable request. The source data underlying Figs. 1a, 1b, 2a, 2b, 4a, 4b, 4c and Supplementary Figs. 2a, 2b, 3a, 3b, 3c,4a, 4b, 5, 7a, 7b, 7c, 7d, 8, 13 are provided as a Source Data file.
