# Supplementary material for: Maximizing sinusoidal channels of HZSM-5 for high shape-selectivity to p-xylene
Source: Nat Commun. 2019 Sep 25;10:4348. doi: 10.1038/s41467-019-12285-4 (PMC6761094; doi:10.1038/s41467-019-12285-4)
Supplement: Supplementary file 1 — Supplementary Information [file 41467_2019_12285_MOESM1_ESM.pdf]

Supplementary Information

Some successful ZSM-5 synthesis but with less *p*-xylene selectivity when pure zeolite was used as catalyst, the synthesis references are classified as follows,

Silicate-1 passivated HZSM-5: China Patent CN102311124B; Molecular Catalysis, 2017, 433, 242-249.

Template-free HZSM-5: Chinese Journal of Catalysis, 2011, 32, 1702-1711; Chinese Journal of Catalysis, 2010, 32, 362-367; Zeolites, 1989, 9, 363-370.

Solvent-free HZSM-5: RSC Advance, 2016, 6, 15816-15820; Microporous and Mesoporous Materials, 2017, 243, 112-118.

Hierarchical HZSM-5: Industrial Engineering Chemical Research 2014, 53, 19471-19478; RSC Advances 2016, 6, 58586-58593; Langmuir, 2004, 20, 8301-8306.

Mixed-template HZSM-5 synthesis: Materials Letters 2012, 74, 115-117; Journal of the Chemical Society, Chemical Communications, 1995, 24, 2473-2670; Zeolites 1982, 2, 313-318; Catalysis Today, 2004, 93–95, 729-734.

Supplementary figures and tables

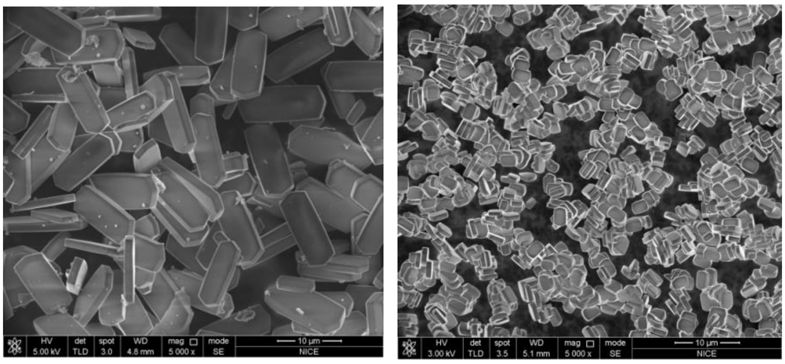

Supplementary Figure 1. SEM images of coffin shape HZSM-5_C1 (left) and HZSM-5_C2 (right)

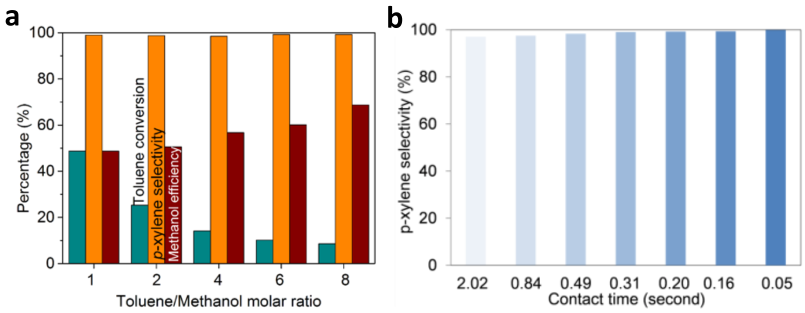

Supplementary Figure 2. Effects of toluene to methanol ratio (mol/mol) (a); and (b) contact time on the performance of HZSM-5_T in methanol-toluene alkylation reaction. Hydrogen flow rate was changed to adjust the contact time. (470 ^{o}C; atmospheric pressure; toluene/methanol (mol/mol) = 6 was co-fed with hydrogen and water)

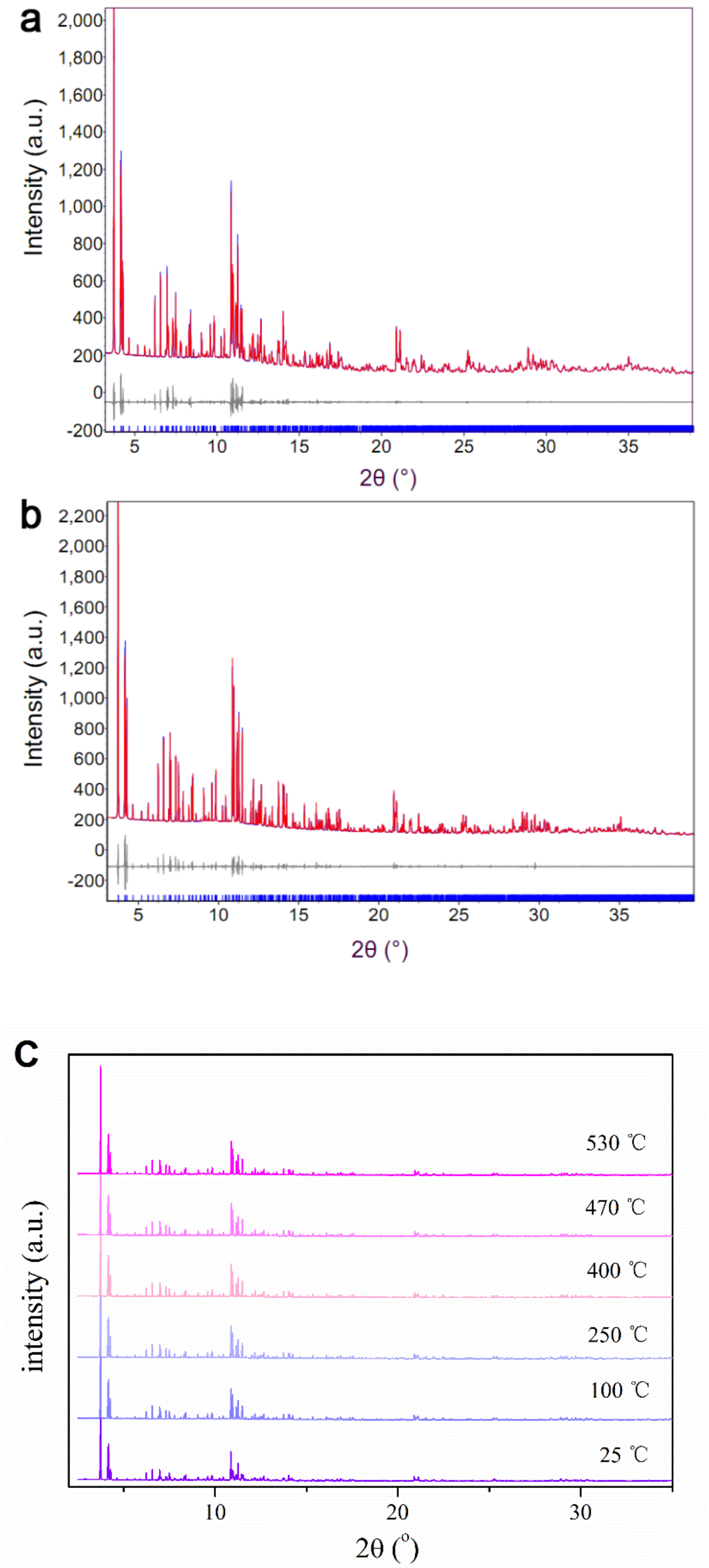

Supplementary Figure 3. Representative Rietveld refinement of synchrotron PXRD patterns of HZSM-5_T zeolite sample at (a) 25 ℃, (b) 470 ℃. (c) synchrotron-based in-situ high temperature PXRD patterns under different temperature. The wavelength was 0.7296 Å.

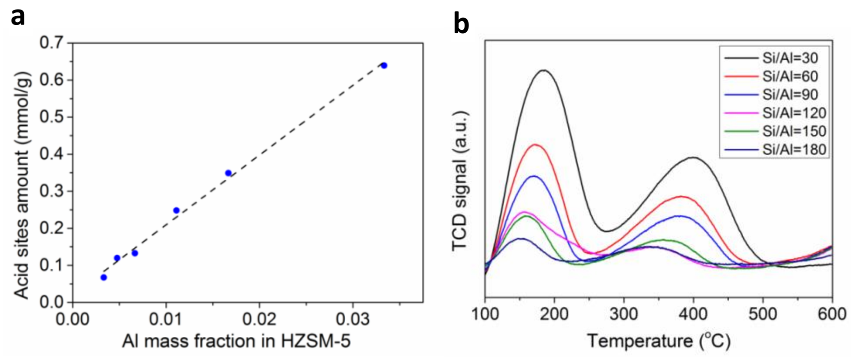

Supplementary Figure 4. Correlations between Si/Al ratios of HZSM-5 and their respective acid sites amount. (a) Relationship between total acid sites amount and Al mass fraction of HZSM-5_T, where the total acid sites amount was obtained from Temperature Programmed Ammonia Desorption (NH_3-TPD) of HZSM-5_T with different Si/Al ratios shown in (b).

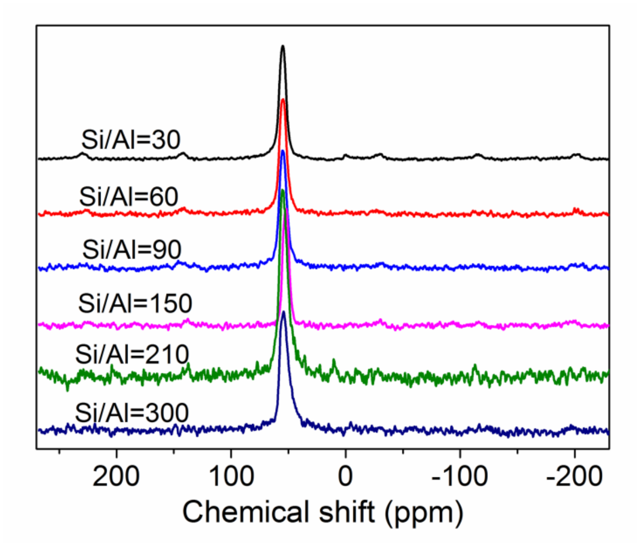

Supplementary Figure 5. ^{27}Al MAS NMR spectra of HZSM-5_T with different Si/Al ratios.

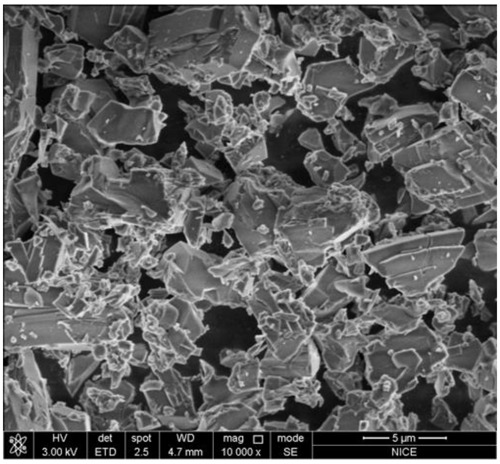

Supplementary Figure 6. SEM image of crushed HZSM-5_T

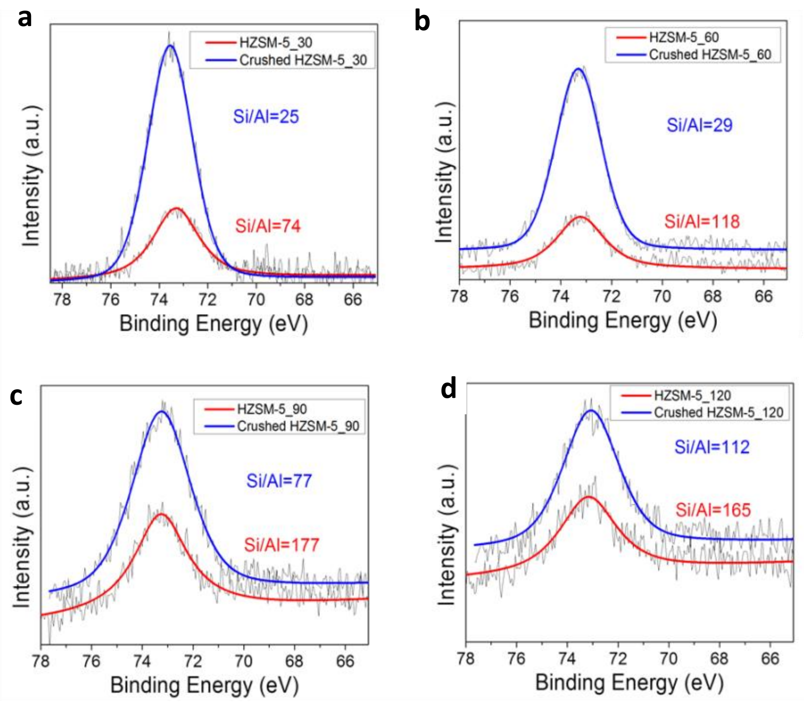

Supplementary Figure 7. Normalized Al *2p* XPS signal of pristine HZSM-5_T crystals with different Si/Al ratios (for example, for HZSM-5_T with Si/Al ratio of 30, it is termed as HZMS-5_30) and their corresponding mechanical crushed HZSM-5 crystals. The Si/Al ratios (determined by XPS) of pristine and crushed zeolites were marked in red and blue respectively.

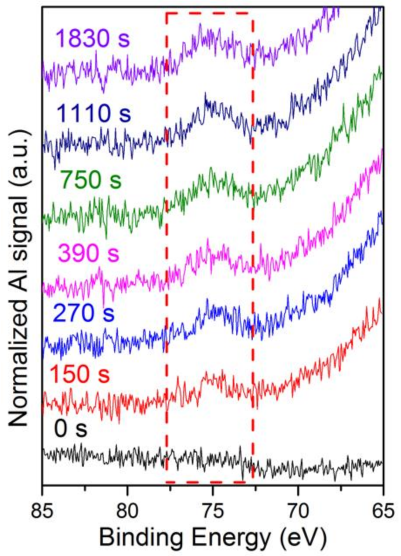

Supplementary Figure 8. XPS depth profile of HZSM-5_C1 showing normalized Al *2p* peak (marked by red dashed rectangle) intensity evolution with Ar-ion sputtering progress

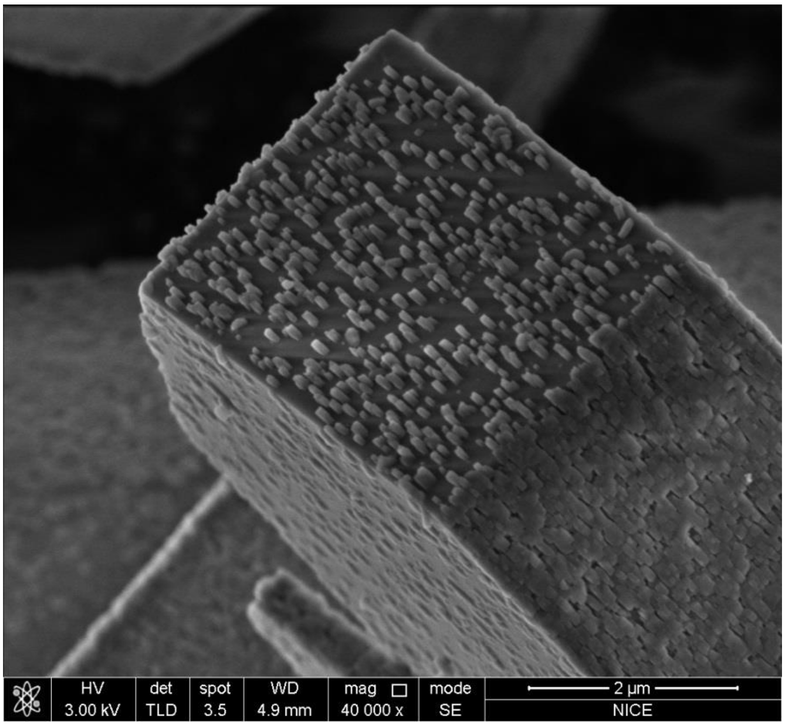

Supplementary Figure 9. Representative SEM image of HZSM-5_T after a surface coating with Silicate-1

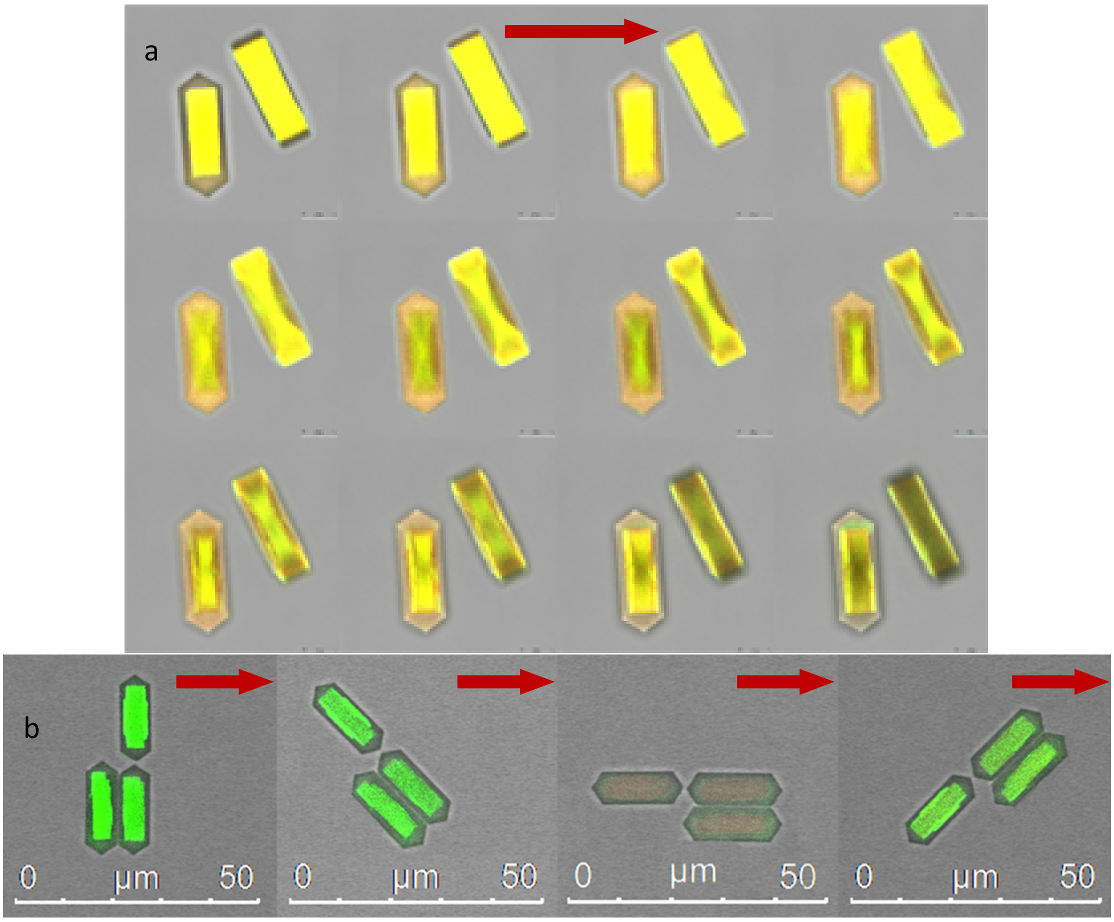

Supplementary Figure 10. Confocal fluorescence microscopy of HZSM-5_T crystals with twinned structures, lying on main crystal (010) facet (left) and (100) facet (right) respectively, with (a) depth scan along *z* axis with 0.5 μm step. It shows the internal diffusion barrier in twinned-structured HZSM-5_T. (b) sample rotation in *xy* plane from 0 ° to 135 ° (anticlockwise) which shows that the strength of fluorescent part of twinned structured HZSM-5_T was relevant to the polarization directions of excitation light. Strongest fluorescence was shown when the channel direction was in line with the direction of polarization light. The arrow in the pictures indicated the direction of polarized light.

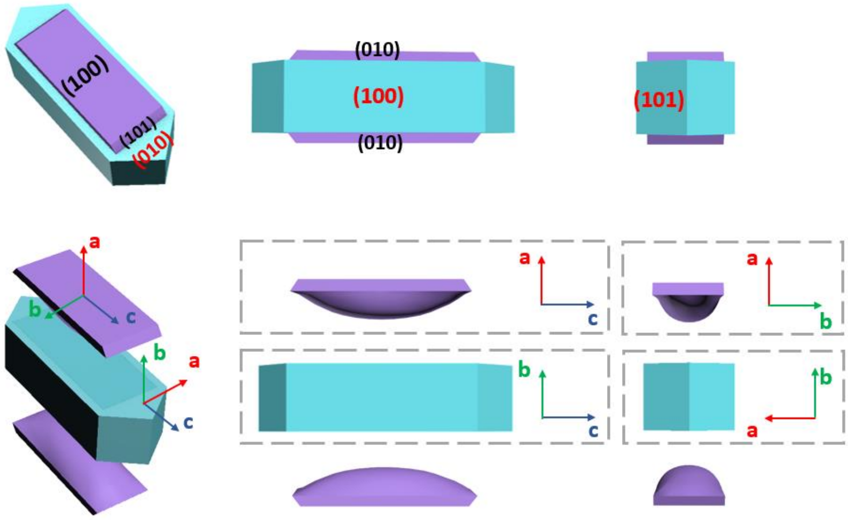

Supplementary Figure 11. Schematic graph of intergrowth HZSM-5_T crystal structure showing its facet orientations, where (100) and (101) facets are the surface with sinusoidal pore openings and (010) facet with straight pore openings.

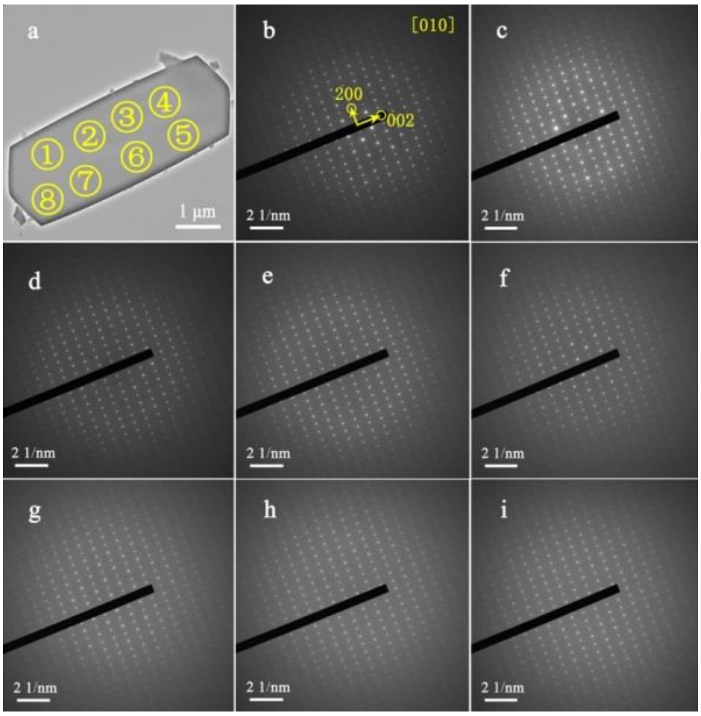

Supplementary Figure 12. Selected-area electron diffraction (SAED) of coffin shape HZSM-5_C1. All the eight selected areas show the same diffraction pattern, implying the single crystal nature of HZSM-5_C1.

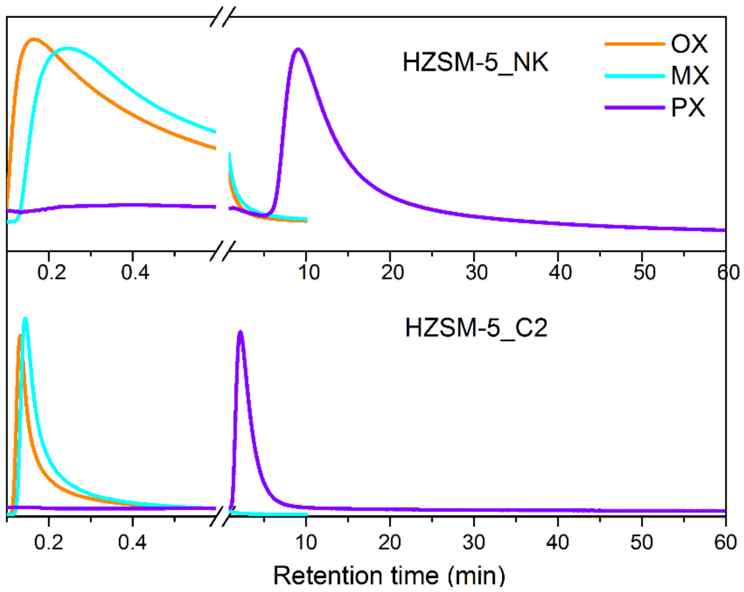

Supplementary Figure 13. Pulse chromatography of xylene isomers (OX: *o*-xylene; MX: *m*-xylene; PX: *p*-xylene) passing through columns packed with HZSM-5_NK and HZSM-5_C2 at 220 ^{o}C.

Supplementary Table 1. Catalytic performance of different catalyst system in methanol toluene alkylation reaction

**Ref.**	**Temp (℃)**	**T/M**	**WHSV**	**C_7H_8 Conv. (%)**	***p*-xylene Sele. (%)**	**Catalyst**	**Si/Al**	**Specific activity (mol. $(mol_{Al})^{-1}.h^{-1}$)**	**TOS (h)**
(15)	400	1	0.62	53.7	50.1	Pure HZSM-5	70	11.3	1
	400	1	0.62	49.9	99.9	SiO_2 modified HZSM-5	70	10.5	1
(14)	400	1	2.1	37.2	98.9	SiO_2 modified HZSM-5	108	40.2	1
(26)	430	8	1.0	6.4	23.3	Pure HZSM-5	40	1.6	-
	430	8	1.0	5.5	31.1	10 %B/HZSM-5	40	1.4	-
	430	8	1.0	0.3	97.8	10 %P/HZSM-5	40	0.07	-
	430	8	2.0	5	>99.9	3 %B/HZSM-5	40	2.54	66.6
(25)	440	8	4.8	10~12	>99.9	B modified	40	13.9	N/A
This study	470	1	7.9	10.2	99.3	Pure HZSM-5	150	144.7	-
	470	2	7.9	12.0	98.9	Pure HZSM-5	150	100.7	-
	470	4	7.9	15.3	99.0	Pure HZSM-5	150	85.0	-
	470	6	7.9	25.3	99.0	Pure HZSM-5	150	74.0	220
(11)	460	2	2	37	27	Pure HZSM-5	13	5.3	-
	460	2	2	21	98	Si-P-Mg-0.3 %Pt modified HZSM-5	13	3.0	500
(17)	400	8	12.4	13.45	25.5	Pure ZSM-5	21	37.4	-
	400	8	12.4	6.75	98.2	13.3 %Si/ ZSM-5	21	18.7	-
(7)	550		3.5	39.8	24.2	Pure ZSM-5	-	-	-
	550		2.1	0.3	67	3.5 %P/HZSM-5	-	-	-
	600		3.5	51.6	24.1	Pure ZSM-5	-	-	-
	600		2.1	0.8	50	3.5 %P/HZSM-5	-	-	-

Supplementary Table 2. The crystallographic details of HZSM-5-T sample from PXRD data refinement under different temperature. The channel radius in HZSM-5-T structure was calculated with the oxygen ionic radius of 1.350 Å.

Temperature (℃).	*a* (Å)	*b* (Å)	*c* (Å)	*β* (°)	Volume	Space group	R_{wp} (%)	Straight $_{min}$ (Å)	Straight $_{max}$ (Å)	Sinusoidal $_{min}$ (Å)	Sinusoidal $_{max}$ (Å)
25	19.8969	20.1043	13.3923	90.423	5357.0188	*P21/n*	6.99	5.229	5.863	5.103	5.731
100	20.1005	19.9193	13.4035	90	5366.6404	*Pnma*	5.41	5.332	5.734	5.443	5.590
250	20.0939	19.9235	13.4040	90	5366.2138	*Pnma*	5.73	5.405	5.673	5.422	5.591
400	20.0795	19.9168	13.3986	90	5358.3968	*Pnma*	6.61	5.407	5.638	5.375	5.552
470	20.0745	19.9148	13.3968	90	5355.8243	*Pnma*	6.69	5.413	5.619	5.353	5.544
530	20.0687	19.9117	13.3943	90	5352.4492	*Pnma*	6.57	5.410	5.591	5.334	5.531

Supplementary Table 3. Effect of surface Si/Al ratios on p-xylene selectivity

Sample	Si/Al ratio		*p*-xylene selectivity (%)	
	Recipe	Surface (XPS)	Before passivation	Passivated by S-1
HZSM-5_30	30	74	62.9	-
HZSM-5_60	60	118	84.0	-
HZSM-5_90	90	177	94.2	97.1
HZSM-5_120	120	165	96.0	98.5
