# Supplementary material for: Maximizing sinusoidal channels of HZSM-5 for high shape-selectivity to p-xylene
Source: Nat Commun. 2019 Sep 25;10:4348. doi: 10.1038/s41467-019-12285-4 (PMC6761094; doi:10.1038/s41467-019-12285-4)
Supplement: Supplementary file 2 — Peer Review [file 41467_2019_12285_MOESM2_ESM.pdf]

Reviewers' comments:

Reviewer #1 (Remarks to the Author):

In “Sinusoidal channels of HZSM-5 for unprecedently high shape selectivity” dr. Ma and coworkers show the ability of highly intergrown ZSM-5 crystals to selectively produce para-xylene from toluene and methanol. The authors support their promising catalytic findings with a wide range of experiments to characterize the material and get insight into the process. The chosen material/reaction system is of general interest to chemists both academically and industrially. The findings described in this manuscript are however not entirely new as the catalyst described by the authors combines two key phenomena of which one has already been explored in great depth i.e. reduction of the surface acidity as these sites do not display shape-selectivity. A surface passivation can be the result of post-treatment or as in this work due to a reverse aluminium gradients. The authors did cleverly exploit a second phenomenon: the difference in molecular transport between the two slightly different pore systems available in ZSM-5 by looking into highly intergrown crystals. These materials are widely described in literature often for fundamental studies while for real catalytic processes they are rarely considered due to a concern of mass-transport limitations.

- the authors want to comment on the crystal size and how it might influence the catalytic performance.

- 'conventional' aluminium zoning occurs with a higher concentration at the surface than at the core. Many have attempted to get the inverse zoning as shown here. The authors want to discuss how synthesis can lead to this ‘inverse’ aluminium distrubtion as well as how to grow such crystals sufficiently large. A strong emphasis should be placed on how to get such crystals with more relevant aluminium contents.

- Related to the previous question, I have my concerns about the low aluminium content of this zeolite and the rather large distance (> 100s of nanometer) over which the molecules should diffuse to reach the zone of catalytically relevant aluminum content. Did the authors consider to take a ZSM-5 sample with decent aluminium content and the commonly encountered intergrown structure and do the typical post synthesis surface passivation? In this case the catalysts should have an even much improved performance.

- the pulse chromatography experiments should be performed at realistic (450C) temperatures as the authors want to show the difference between the different Xylenes and here the temperature dependent pore-diameter (see figure 1) plays an important role. And how do the authors explain the observed trend for the main desorption peak of o-Xylene and m-Xylene in the two batches (that is: o-Xyl_C1 < o-Xyl_T ~= m-Xyl_T < m_Xyl_C1 ?

- Again related to the previous point, the pore diameter is highly temperature dependent. More information needs to be provided on how this was determined. In the text confusion between the IZA defined pore diameter and the measured/real pore diameter exists.

- The difference between the two samples for p-Xylene desorption is very important (at least 100 mins more of retention for the intergrowth crystal !) while the changes in conversion/selectivity is not so drastic (same conversion and "only" an increase of 17% in selectivity towards p-Xylene). Thus the explanation provided by the authors is not very clear to me. This chromatography experiments (at relevant temp) should be conducted for all ZSM-5 materials discussed.

- Now, I would love to be convinced of an efficient pore-induced selectivity in this case, but the evidences are not the most convincing, some left me confused as you could see above. So let's play the devil's advocate and reconsider the crystal size.
HZSM5_C1 and HZSM5_C2 have a fairly similar Si/Al (135 & 150) and only differ in size (C1 is 5-10x

bigger than C2). This leads to a drop of selectivity to p-Xylene from 82 to 31%
Now HZSM5_C1 and HZSM-T also have a similar Si/Al (135 & 148), but this time T crystals are sligthly bigger but also much more thicker than C1 ones (from 1-2 um to 5-8um). The authors observe an increase of selectivity to p-Xylene from 82 to 91%. Could it be due to the crystal size rather than its pore structure?

About the form/text:
- It could be interesting to include the Table S2 in the manuscript
- Some typos: "modifier" line 47 ; "differentiate" line 53
- I don't agree with the sentence "big straight channels and small sinusoidal channels" in the abstract. Of course there is a size difference, but not sufficient enough to use the big/small comparison in my opinion
- Weird sentence starting end of line 106: "we believe that HZSM-5_T indeed has acid sites density gradient from surface to core, with very few acid sites on the external shell of the crystals to avoid the non-shape selective reactions, while a Si/Al ratio around 100 is reached beneath".
- Typo (?) in the SI @Figure S7. Based on the manuscript it should be HZSM-5_C1
- figure S8 I believe irresponsive is not correct. There is a clear response to the polarization.

Overall, this is a potentially interesting scientific work, which with further convincing evidence could be relevant to the wider chemical readership of Nature Communication

Reviewer #2 (Remarks to the Author):

This manuscript describes a intergrown MFI crystals and their use for p-xylene formation. The evidence of the contribution of the structure to the high p-xylene selectivity is not enough. The p-xylene selectivity often reaches 100% when the crystal size is large because of the diffusion limitation. The crystal size of HZSM-5-T in this study is larger than 20 micrometer which is larger than HZSM-5-C1 and HZSM-5-C2. The high para-xylene selectivity is reasonable results without considering the intergrown structure.

Reviewer #3 (Remarks to the Author):

It is an interesting work reporting on development of MFI catalysts with controlled morphology to improve the selectivity to p-xylene in toluene methylation reaction. The idea is to synthesize ZSM-5 catalyst with the crystal plane of (100) on the external surface, which opens sinusoidal channels. Due to the slightly small sinusoidal channel compared to the straight channel, the p-xylene selectivity is largely improved. In the study, such structure has been achieved by using a intergrowth twin crystal structure in ZSM-5 catalysts. The authors have provided a series of characterization to demonstrate the significantly improved selectivity to p-xylene is due to the large particle size, inert external surface and intergrowth twin structure. The effects of large particle size and inert external surface on the p-xylene selectivity have been extensively studied in the past. The novelty of the study is mainly coming from the intergrowth twin structure control. Control of the intergrowth twin structure is a challenge. In the past, several studies have shown the intergrowth twin structures in large ZSM-5 catalysts and found the intergrowth structure is sensitive to the synthesis condition. In most cases, it is difficult to synthesize ZSM-5 crystals with the same intergrowth structures. Several different intergrowth structures have been reported in the literature (e.g., J. AM. CHEM. SOC. 2008, 130, 5763–5772 and Nature Materials, 8(12), 959–965.). The catalytic result reported in the study is very promising, however, the synthesis work is not convincing. It is important to show how such a unique intergrowth structure has been achieved. The synthesis method reported in the SI is very brief even without the composition. n-Butylamine was used in the synthesis with TPABr, which is not a conventional method to make ZSM-5 material. The lack of synthesis information

might be due to patent related issues. However, it is needed for the work. In addition, discussion on the effect of synthesis parameters on the morphology is also critical for the work in order to provide a fundamental understanding for the material. Some additional technical comments.

1. What is the information that we can learn from the confocal fluorescence microscopy study? I feel the study only shows the presence of internal diffusion barrier, but no information about the intergrowth structure. The data should be similar to the sample of C1.
2. Al source usually plays critical role in zeolite synthesis. What is the Al source used in the synthesis and what is its effect on the morphology?
3. The pulsed chromatography test reported in Figure 4 is confusing. What is the amount of o, m and p-xylene was injected into the zeolite column? Is all p-xylene adsorbed in the zeolite as shown in Figure 4b. Also for Figure 4c, can we calculate the diffusivity of m-xylene and compared to literature data? Can we do the same measurement using p-xylene?

Response to the comments of reviewers

Reviewer #1: In "Sinusoidal channels of HZSM-5 for unprecedently high shape selectivity" Dr. Ma and coworkers show the ability of highly intergrown ZSM-5 crystals to selectively produce para-xylene from toluene and methanol. The authors support their promising catalytic findings with a wide range of experiments to characterize the material and get insight into the process. The chosen material/reaction system is of general interest to chemists both academically and industrially. The findings described in this manuscript are however not entirely new as the catalyst described by the authors combines two key phenomena of which one has already been explored in great depth i.e. reduction of the surface acidity as these sites do not display shape-selectivity. A surface passivation can be the result of post-treatment or as in this work due to a reverse aluminium gradients. The authors did cleverly exploit a second phenomenon: the difference in molecular transport between the two slightly different pore systems available in ZSM-5 by looking into highly intergrown crystals. These materials are widely described in literature often for fundamental studies while for real catalytic processes they are rarely considered due to a concern of mass-transport limitations.

Question 1. The authors want to comment on the crystal size and how it might influence the catalytic performance.

Answer 1. We thank the reviewer for the comment. Following the suggestion of the reviewer, the influence of crystal size on shape-selectivity has been discussed in lines 51~53 "Except for modifications to HZSM-5 catalyst, *p*-xylene selectivity can also be enhanced to some extent by maximizing xylene isomers' diffusion resistance in HZSM-5 pores, for example, large HZSM-5 crystals always show higher selectivity to *p*-xylene with elongated transport length" and 97-99 "Larger zeolite crystal was one of the reasons since size increase always means longer diffusion path. Niwa et al[32] has found there was relevance between shape-selectivity and HZSM-5 crystal size, i.e, large HZSM-5 crystals generally show higher selectivity to *p*-xylene than small ones.". Yes, for the shape-selective process of *p*-xylene, Niwa et al (*Journal of Catalysis 173, 433–439 (1998), Ref. 33*) has found there was relevance between shape-selectivity and HZSM-5 crystal size. In this paper, to exclude the influences of size and external surface acidity on shape selectivity, we tried to use a similar sized, surface aluminum poor HZSM-5_C1 (SEM in Figure S1) with unobstructed channels (SAED in Figure S11) as a comparison, although this zeolite couldn't have the same three dimensions in both a, b and c axis as HZSM-5_T. HZSM-5_C1 only showed moderate selectivity 81.9%, a comparable value to that of Niwa et al, to *p*-xylene. Thus, except for HZSM-5 crystal size and external surface acidity, it is concluded that there are some other factors that make intergrowth HZSM-5_T stands out with selectivity up to 99.3%.

Question 2. 'Conventional' aluminum zoning occurs with a higher concentration at the surface than at the core. Many have attempted to get the inverse zoning as shown here. The authors want to discuss how synthesis can lead to this 'inverse' aluminum distribution as well as how to grow such crystals sufficiently large. A strong emphasis should be placed on how to get such crystals with more relevant aluminum contents.

Answer 2. We also screened a large quantity of synthesis methods to obtain HZSM-5 with less external surface acid sites. Generally, these synthesis methods would result in higher

concentration of acid sites on the surface. The one we reported here was an exception. The double template TPABr and n-butylamine system is supposed to be the main reason for the formation of large crystal. Although the use of double template was not conventional in HZSM-5 synthesis, it had been applied to the synthesis of another large size MFI zeolite, TS-1 (Wang et al, Catalysis Today, 2002, 74(1-2):65-75), Guo et al, Studies in Surface Science and Catalysis, 1997, 112, 499-508). For the inverse aluminum distribution, we speculated the quick hydrolysis of Al precursor with the addition of TPABr could form the very initial Al-rich nucleus (visible when the clear TPABr solution and NaAlO2 solution was mixed to form turbid gel), and the following crystallization of Si precursors around the already formed Al-rich nucleus may result in the inverse aluminum zoning. The uneven Al distribution may also relate to the large crystal obtained from the synthesis. We will try to do more experiment to address this difficult problem in the future.

Question 3. Related to the previous question, I have my concerns about the low aluminum content of this zeolite and the rather large distance (> 100s of nanometer) over which the molecules should diffuse to reach the zone of catalytically relevant aluminum content. Did the authors consider to take a ZSM-5 sample with decent aluminum content and the commonly encountered intergrown structure and do the typical post synthesis surface passivation? In this case the catalysts should have an even much improved performance.

Answer 3. We thank the reviewer for the suggestion. In our research, we did passivate a few intergrowth HZSM-5 samples, which have lower Si/Al ratios ranged from 30 to 100, with a method previously reported (*Molecular Catalysis, 433, 242-249 (2017)*), namely coating HZSM-5 of intergrowth structure with a thin layer of Silicate-1. These pristine HZSM-5 has a para-selectivity ranged from 92%-95% and surface passivation could enhance the selectivity to about 97%, but still could not reach 100%. We think beside the inverse aluminum gradient (especially the Al content in the near-surface area), the channel structure of our catalysts is equally important for the ultra-high selectivity to p-xylene.

Question 4. The pulse chromatography experiments should be performed at realistic (450C) temperatures as the authors want to show the difference between the different Xylenes and here the temperature dependent pore-diameter (see figure 1) plays an important role. And how do the authors explain the observed trend for the main desorption peak of o-Xylene and m-Xylene in the two batches (that is: o-Xylne_C1 < o-Xylene_T ~= m-Xylne_T < m_Xylene_C1 ?

Answer 4. We thank the reviewer for the suggestion. An explanation to the pulsed chromatography has been appended to line 181-184 to help understand this technique. The test temperature was set at 470 °C because of the best catalytic reactivity for toluene methylation. For the temperature setting of the pulsed chromatography experiments, a temperature close to the reaction temperature 470 ^{o}C would be ideal. However, a lower temperature was used to ensure a minimum xylene isomerization reaction, especially for p-xylene and o-xylene (Collins et al, *The Canadian Journal of Chemical Engineering,* 61(1), 29-35 (1983), although the acid-sites poor surface of HZSM-5_T and HZSM-5_C1 may help to reduce the possibility of isomerization to some extent. Moreover, the conversion of xylene isomerization increases at higher temperature (Li et al, *Industrial Engineering and Chemical Research*, 31(1), 187-192 (1992)). In our experiment, we found the conversion of xylene isomerization can be as high as 50% at 470 ^{o}C. For this reason,

the isomerization of each HZSM-5 sample was measured before the pulsed chromatography test and 220°C is a moderate temperature to maintain low xylene isomerization reaction (less than 2%). The temperature for the pulsed chromatography experiment was also limited by the GC oven. At higher temperatures, the size-exclusion capability of HZSM-5 was expected to be even better since both the maximum size of sinusoidal channel and straight channel were getting smaller with temperature increase (Figure 1a).

Sorry for the confusion in Figure 1 caused by the abscissa scale difference, which made it look like o-Xylne_C1 < o-Xylene_T. It has been corrected in the revised version. Actually, o-xylne_C1 = o-xylene_T, which was supposed to be a result that o-xylene has the biggest size (of the three xylene isomers) and can hardly ("hardly" but not "absolutely not", otherwise a sharp peak rather than an expanded elution peak would be found) enter either the pores of HZSM-5_T or HZSM-5_C1, and thus has the same and the least retention time for both HZSM-5_T and HZSM-5_C1. m-xylene was considered to be unable to enter the pores of HZSM-5_T (same elute time as o-xylene), but partially into the pores of HZSM-5_C1 (a little higher retention time than o-xylene), thus a lower retention time was observed over HZSM-5_T than coffin shape HZSM-5_C1. Meanwhile, the narrower peaks over HZSM-5_T than HZSM-5_C1 also indicated m-xylene and o-xylene are less absorbed by the pores of HZSM-5_T.

Question 5. Again related to the previous point, the pore diameter is highly temperature dependent. More information needs to be provided on how this was determined. In the text confusion between the IZA defined pore diameter and the measured/real pore diameter exists.

Answer 5. As suggested, information about the Rietveld refinement of the synchrotron X-ray Diffraction data has been appended to SI section "Synchrotron XRD test" and Figure S3, as well as Table S2. The pore diameter difference between IZA defined and real diameter here was caused by different synthesis conditions or sample states. The pore diameter defined by IZA was based on an as-prepared Na-ZSM-5 without calcination, while in this paper, the pore size is based on calcined HZSM-5 samples by considering the diameter of oxygen atoms as 2.7Å. In fact, any change of the composition of zeolite would have minor influence on the unit cell and atomic positions, and thus the pore diameter may change accordingly.

Question 6. The difference between the two samples for p-Xylene desorption is very important (at least 100 mins more of retention for the intergrowth crystal!) while the changes in conversion/selectivity is not so drastic (same conversion and "only" an increase of 17% in selectivity towards p-Xylene). Thus the explanation provided by the authors is not very clear to me. This chromatography experiments (at relevant temp) should be conducted for all ZSM-5 materials discussed.

Answer 6. This is a nice comment. As discussed in Answer 4, a relatively lower temperature (220 ^{o}C) is used for the pulsed chromatography to prevent the isomerization of xylenes at elevated temperature, since it was zeolite catalysts packed in the column for the test. We also append the manuscript accordingly in line 180-184 "were tested by pulsed chromatography and by applying the theory of size exclusion column, i.e., molecules that can't enter the pore would elute first (the minimum retention time) because they have the least volume to move, while molecules that can enter the pores of HZSM-5 would elute the last (the maximum retention time) due to more volume to travel." to make the pulsed chromatography more easily to be understood.

As suggested, the pulsed chromatography results of the other two surface-aluminium-rich HZSM-5 samples, HZSM-5_C2 and HZSM-5_NK, have been appended in Figure S12 with appended explanation "For the other two surface aluminium rich HZSM-5 samples, HZSM-5_NK also showed incapability of preventing the entry of *m*-xylene into its pores, while HZSM-5_C2 seemed to have difficulty to permit the entry of even p-xylene, since it flow out of the zeolite column shortly after the injection with a retention time of only 2.17 minutes, as shown by the pulsed chromatography result in Figure S12.".

It should be noted both pore size and external surface acid sites are important factors that influence the p-xylene selectivity in toluene alkylation. The shape-selectivity enhancement by pore size narrowing can only be shown when the influence of external acid sites has been excluded. Both sample HZSM-5_C2 and HZSM-5_NK have rich acid sites on their external surface (Table 1), and even under 250°C, these two zeolite showed noticeable p-xylene and o-xylene isomerization (2%~5%). Under this circumstance, it was supposed the secondary isomerization of p-xylene dominated the very low shape-selectivity to p-xylene in toluene alkylation.

The conversion of toluene was considered to be dependent on the accessible acid sites of HZSM-5, so it is almost the same for all the catalysts with similar Si/Al ratios. Though the increase from 82% to 99.3% in selectivity towards p-xylene is not drastic, 17.3% is still considerable as the selectivity of HZSM-5_T has been very close to 100%, which could be attributed to the HZSM-5_T sinusoidal channels. We believe the sinusoidal channels induced superior shape-selectivity could be better demonstrated with molecules have even smaller difference than xylene isomers. Meanwhile, unlike modified HZSM-5 over which high p-xylene selectivity can only be obtained with a very short reactant-catalyst contact time, the superior selectivity of HZSM-5_T toward p-xylene could be reached on a broad range of contact time and toluene-to-methanol ratios.

Question 7. Now, I would love to be convinced of an efficient pore-induced selectivity in this case, but the evidences are not the most convincing, some left me confused as you could see above. So let's play the devil's advocate and reconsider the crystal size. HZSM5_C1 and HZSM5_C2 have a fairly similar Si/Al (135 & 150) and only differ in size (C1 is 5-10x bigger than C2). This leads to a drop of selectivity to p-Xylene from 82 to 31%. Now HZSM5_C1 and HZSM-T also have a similar Si/Al (135 & 148), but this time T crystals are slightly bigger but also much more thicker than C1 ones (from 1-2 um to 5-8um). The authors observe an increase of selectivity to p-Xylene from 82 to 91%. Could it be due to the crystal size rather than its pore structure?

Answer 7. We thank the reviewer for his/her very careful consideration. Indeed for a system with the contribution of different factors, it is not easy to isolate out the role of each player. A 17.3% p-xylene selectivity change, from 82% to 99.3% was observed over HZSM-5_T, when compared to HZSM-5_C1. Here, the core contribution of external surface acid sites has been excluded since both the two zeolites are surface-aluminum-poor.

The para-selectivity, which is caused by the diffusion resistance difference between xylene isomers, can be improved by either zeolite crystal size increase or pore narrowing. We do agree the increase of thickness, that is, longer diffusion paths for xylene isomers, could improve p-xylene selectivity. However, when zeolite's pore size is comparable to m-xylene, a small decrease in pore size would lead to a large decrease in diffusivity (Bu et al., *J. Phy. Chem. C,*

121(1), 500–510 (2017)). In Figure 4c, we found that when the thickness (length of b-axis of HZSM-5) was taken into consideration, i.e., the calculation of diffusion coefficient rather than the diffusivity, the diffusion coefficient of m-xylene calculated from Figure 4c is around 2 times higher over HZSM-5_C1 than over HZSM-5_T. Meanwhile, Figure 4a and Figure 4b showed m-xylene could partially enter the pores of HZSM-5_C1 but hardly enter the pores of HZSM-5_T. So in this work we focus on discussing the influence of the pore structure, where the results showed the pores size change did affect the diffusion coefficient. Surely, the exact quantitative role of each factor need further research and open to discussion.

Question 8. About the form/text:

- It could be interesting to include the Table S2 in the manuscript
- Some typos: "modifier" line 47 ; "differentiate" line 53
- I don't agree with the sentence "big straight channels and small sinusoidal channels" in the abstract. Of course there is a size difference, but not sufficient enough to use the big/small comparison in my opinion
- Weird sentence starting end of line 106: "we believe that HZSM-5_T indeed has acid sites density gradient from surface to core, with very few acid sites on the external shell of the crystals to avoid the non-shape selective reactions, while a Si/Al ratio around 100 is reached beneath".
- Typo (?) in the SI @Figure S7. Based on the manuscript it should be HZSM-5_C1
- figure S8 I believe irresponsive is not correct. There is a clear response to the polarization.

Answer 8. Thanks for pointing out the typos and language problems in the text. They have been refined in the revised version.

(1). Table S2 has been included in the manuscript as Table 1.

(2). Typos inline 47 and line 53 has been corrected.

(3)"big straight channels and small sinusoidal channels" has been changed to "different sized straight channels and sinusoidal channels", now in line 20.

(4) Line l06 has been changed to "we believe that HZSM-5_T indeed has acid sites density gradient from surface to core, with very few acid sites on the external surface of the crystals to avoid the non-shape selective reactions, and most of the acid sites reside in the inner core." Now in line 111-113.

(5) Typo in SI @Figure S7 (now Figure S8) was changed.

(6) Sorry for the wrong expression of Figure S8 (now Figure S9) in the previous version, we intent to explain the parts that can be illuminated were irrelevant to the polarization direction. Yes, the strength of the fluorescent part is strongly related to the direction of polarization. It has been changed to the correct expression in SI line 108 as "which shows that the strength of fluorescent part of twinned structured HZSM-5_T was relevant to the polarization directions of excitation light."

Reviewer #2

This manuscript describes a intergrown MFI crystals and their use for p-xylene formation. The evidence of the contribution of the structure to the high p-xylene selectivity is not enough. The p-xylene selectivity often reaches 100% when the crystal size is large because of the diffusion

limitation. The crystal size of HZSM-5-T in this study is larger than 20 micrometer which is larger than HZSM-5-C1 and HZSM-5-C2. The high para-xylene selectivity is reasonable results without considering the intergrown structure.

Answer: Thanks for the comments. Figure 4a and Figure 4b showed the distinct adsorption behaviors of m-xylene over HZSM-5_C1 and HZSM-5_T. These results show the pore size change is also an important contributing factor that affects p-xylene selectivity. In the same time, the crystal size factor has been taken into consideration when measuring the diffusion coefficient in Figure 4c, which still shows the difference when the size factor was excluded.

Reviewer #3

The catalytic result reported in the study is very promising, however, the synthesis work is not convincing. It is important to show how such a unique intergrowth structure has been achieved. The synthesis method reported in the SI is very brief even without the composition. n-Butylamine was used in the synthesis with TPABr, which is not a conventional method to make ZSM-5 material. The lack of synthesis information might be due to patent related issues. However, it is needed for the work. In addition, discussion on the effect of synthesis parameters on the morphology is also critical for the work in order to provide a fundamental understanding for the material.

Answer: Following the suggestion of the reviewer, we have rewritten the synthesis section in the supporting information. Now the synthesis steps as well as the reaction composition were listed in detail in the SI. For the intergrowth structure, it is indeed very difficult to get the clear mechanism. We speculate the combination of TPABr and n-butylamaine is important for the construct of the intergrowth structure. Although the use of double template was not conventional in HZSM-5 synthesis, it had been applied to the synthesis of another large size MFI zeolite, TS-1 (Wang et al, Catalysis Today, 2002, 74(1-2):65-75), Guo et al, Studies in Surface Science and Catalysis, 1997, 112, 499-508). We will try to do more experiment to address this difficult problem in the future.

Question 1. What is the information that we can learn from the confocal fluorescence microscopy study? I feel the study only shows the presence of internal diffusion barrier, but no information about the intergrowth structure. The data should be similar to the sample of C1.

Answer 1: We thank the reviewer for the constructive comment. Yes, the confocal fluorescence microscopy study gave us a much clearer profile of the intergrowth we observed in Figure 3c by TEM. With its depth scan function of fluorescence microscopy, we were able to obtain the information of the intergrowth layer by layer (Figure S9a) and rebuild the intergrowth crystal (Figure S10), while this could be hard for TEM since TEM gives more fine details. Moreover, the part embedded in the parent HZSM-5 has long been supposed to be a pyramid shape without internal connections, now proved to be an oval hemisphere. However, due to the resolution of confocal fluorescence microscopy, we were unable to disclose detailed structure of the barrier. Meanwhile, because the crystal only gave strong fluorescence when its channel direction was in line with the direction of polarized laser, it is easy to discern the direction of zeolite channels, as well as the existence of twin-crystals.

The structure of HZSM-5_C1 is different from HZSM-5_T with unobstructed pore openings, as shown by the SAED analysis in Figure S11, no internal barrier was found over HZSM-5_C1. We also have confocal fluorescence microscopy (not shown in the manuscript) showing HZSM-5_C1 was single crystal only.

Question 2. Al source usually plays critical role in zeolite synthesis. What is the Al source used in the synthesis and what is its effect on the morphology?

Answer 2. Nice comment. Aluminum source did play a key role in ZSM-5 synthesis. The aluminum source used in this research is $NaAlO_2$. We also tried aluminum sulfate and aluminum isopropoxide as the aluminum sources but failed to get the designated structure. The role of different aluminum sources was not studied in detail yet. However, we found factors such as PH, H_2O/SiO_2 ratio, crystallization temperature, Si/Al ratio as well as the way of crystallization (steric or dynamic autoclaves) are critical to the size control of the zeolite crystals, while crystallization time only have minor influence on ZSM-5 crystal size.

Question 3. The pulsed chromatography test reported in Figure 4 is confusing. What is the amount of o, m and p-xylene was injected into the zeolite column? Is all p-xylene adsorbed in the zeolite as shown in Figure 4b. Also for Figure 4c, can we calculate the diffusivity of m-xylene and compared to literature data? Can we do the same measurement using p-xylene?

Answer 3. The pulsed chromatography was adapted from size-exclusion chromatography, i.e., with HZSM-5 as the porous material to absorb molecules of different sizes, molecules that can't enter the pore would elute first (the minimum retention time) because they have the least volume to move, while molecules that can enter the pores of HZSM-5 would elute the last (the maximum retention time) due to more volume to travel. Ideally, the pulsed chromatography experiment was to be tested at a temperature close to the toluene alkylation temperature. However, HZSM-5 is also a good catalyst for the isomerization of xylene, and xylene conversion increases at elevated temperature (Li et al, *Industrial Engineering and Chemical Research*, 31(1), 187-192 (1992); Guisnet et al. *Microporous and Mesoporous Materials*, 35-36, 47–59 (2000); Collins et al, *The Canadian Journal of Chemical Engineering,* 61(1), 29-35 (1983)). In our experiment, we found the conversion of xylene isomerization can be as high as 50% at 470 ^{o}C. For this reason, the isomerization of each of the HZSM-5 sample was measured before the pulsed chromatography test and 220°C is a moderate temperature to maintain low xylene isomerization reaction (less than 2%).

In this experiment, the amount of *o*, *m* and *p*-xylene injected into the GC inlet was controlled to 0.1 μL, and the split ratio of the inlet was 120, so the amount of xylene injected into the zeolite column was around 8.3×10^{-4}μL. In Figure 4b, *p*-xylene was completely absorbed by HZSM-5_T and could not elute from the zeolite column at the test temperature, which was considered to be a result of the lower diffusion rate of p-xylene via sinusoidal channels when compared with straight channels (Bu et al, *J. Phys. Chem. C.* 2017, 121, 500-510).

The room temperature diffusion coefficient of m-xylene calculated from Figure 4c was 1.0×10^{-15} m^2s^{-1} and 1.9×10^{-15} m^2s^{-1} for HZSM-5_T and HZSM-5_C respectively. This value is comparable to the experiment value of 1.0×10^{-15} m^2s^{-1} ~ 1.0×10^{-16} m^2s^{-1}determined by Corma et al

(*J. Phys. Chem.*, 99, 14064-14071 (1995)) and 0.9×10^{-15} m^2s^{-1} determined by Choudhary et al (Zeolites, 6(3), 206–208 (1986)). The same method can also be applied to determine the diffusivity of p-xyelne. In our research, the diffusion coefficient of p-xylene determined by this method (data not shown in the paper) was 3.4×10^{-13} m^2s^{-1} for HZSM-5_C and 1.2×10^{-13} for HZSM-5_T respectively, which is also comparable to the literature value 4×10^{-13} m^2s^{-1} reported by Han et al (*Ind. Eng. Chem. Res*. 38, 3172−3175(1999)).

REVIEWERS' COMMENTS:

Reviewer #1 (Remarks to the Author):

In the revised version the authors have provided a very detailed response to the reviewers, the changes to the manuscript are however very/too succinct and the language used in these changes is sloppy e.g. line 97 'Large zeolite crystal was one …' should be 'The large zeolite crystal is one…'; SI line 3: aquous, line 5: deioned to name a few. Further, it is still not clear from the provided data to which extent different factors (size, low Al content on the surface, pore orientation) create the shown high selectivity. Also, I still feel there is a problem with the samples not being industrially relevant, and not even very typical HZSM-5. But overall I think the scientific results are interesting to the researchers in the field. In my opinion, the authors should still provide quite some changes to the manuscript to make it more readable. I have structured them according to my original questions.

Original question 2:
Link between synthesis and aluminium zoning. The authors report to have screened many different synthesis procedures of which only the one in the manuscript is the only one that gave this inverse zoning. The researchers in the field would benefit if the authors include all of these 'large quantity' of synthesis methods that have been tested and the result that was obtained as it will avoid other researchers to unnecessarily try them again. Here the authors should also add the reasoning to why they think this synthesis is different cfr. response letter.

Original question 3:
It is very interesting to learn that the authors also have tried to do surface passivation, but I am puzzled why this is not included in the manuscript or SI. It should be added to the discussion also mentioned here above.

Original question 4:
1) A main source of concern in the original manuscript was related to the pulsed chromatography experiment and the used temperature. The authors justify this by pointing out that at elevated temps the conversion between the different xylenes is too large, but the discussion is not clearly included in the manuscript.
2) in supporting info the authors now discuss the 2 other zeolite samples (figure s12), it is very confusing that the colors of the different xylene isomers is different than that in the main article figure 4. This should be modified. Also the manuscript would have benefited from a more in depth discussion, especially the new data provided in SI. Although I understand the communication format does not allow to extend too much the discussion, I am sure it is essential for the reader, who will not have access to the rebuttal letter.

Figure s8: the link between the XPS depth profile (time) to depth in nm should be shown as it is highly relevant.

Typo:
Fluorephore => fluorophore

Reviewer #3 (Remarks to the Author):

The group has added significant amount of work to address my concern from the previous review. In particular, adding the synthesis method is crucial for the work. Apparently, adding n-butylamine in the synthesis solution has some effects on the morphology and the crystal structure of ZSM-5 catalyst. I hope the group can study the effects of n-Butylamine on the morphology in future and address the intriguing structure-property relationship and how to control it using additional organic molecules.

Response to the comments of reviewers

Reviewer #1:

Question 1. In the revised version the authors have provided a very detailed response to the reviewers, the changes to the manuscript are however very/too succinct and the language used in these changes is sloppy e.g. line 97 'Large zeolite crystal was one …' should be 'The large zeolite crystal is one…'; SI line 3: aquous, line 5: deioned to name a few. Further, it is still not clear from the provided data to which extent different factors (size, low Al content on the surface, pore orientation) create the shown high selectivity. Also, I still feel there is a problem with the samples not being industrially relevant and not even very typical HZSM-5. But overall I think the scientific results are interesting to the researchers in the field. In my opinion, the authors should still provide quite some changes to the manuscript to make it more readable. I have structured them according to my original questions.

Answer 1. We thank the reviewer for the very constructive comment. Following the suggestion of the reviewer, we have checked the language problems to ensure the high quality of the manuscript. The modifications are highlighted in the main article and listed here as follows,

Line 24 and line 26, "was" and "showed" have been changed to present tense "is" and "shows" to conform to abstract rules.

Line 101 "Large zeolite crystal was one of the reasons…" has been changed to "The larger zeolite crystals were one of the reasons…"

Supplementary Information typos of "aquous" and "deioned" have been changed to "aqueous" and "deionized", now in Methods section line 256 and line 258.

Question 2. Link between synthesis and aluminum zoning. The authors report to have screened many different synthesis procedures of which only the one in the manuscript is the only one that gave this inverse zoning. The researchers in the field would benefit if the authors include all of these 'large quantity' of synthesis methods that have been tested and the result that was obtained as it will avoid other researchers to unnecessarily try them again. Here the authors should also add the reasoning to why they think this synthesis is different cfr. response letter.

Answer 2. Thanks for the comment. As suggested, the reference synthesis methods we tried have been appended to the Supplementary Information. Meanwhile, we also appended our reasoning of the Al zoning difference in main article line 130-135 with the description "The zoned Al distribution was first discovered by Balloom et al[39] and Al zoning seemed to be more common with large ZSM-5 crystals over which Al rich rims were frequently observed. It is sure the Al zoning has close relationship with the recipe of zeolite synthesis and the procedures. Although the exact reasons lead to this reverse Al zoning is not clearly known yet, the large crystal reason can be excluded here since both Balloom et al[39] and Groen et al[40] reported Al rich ZSM-5 crystal rim with crystal size ranged from 25 μm to 50 μm."

Question 3. It is very interesting to learn that the authors also have tried to do surface passivation, but I am puzzled why this is not included in the manuscript or SI. It should be added to the discussion also mentioned here above.

Answer 3. Thanks for the comment. As suggested, the results of passivated HZSM-5 with different Si/Al ratios have been appended in line 135-141, Supplementary Information Figure 9 and Supplementary Information Table 3.

Question 4

1) A main source of concern in the original manuscript was related to the pulsed chromatography experiment and the used temperature. The authors justify this by pointing out that at elevated temps the conversion between the different xylenes is too large, but the discussion is not clearly included in the manuscript.

2) in supporting info the authors now discuss the 2 other zeolite samples (figure s12), it is very confusing that the colors of the different xylene isomers is different than that in the main article figure 4. This should be modified. Also the manuscript would have benefited from a more in depth discussion, especially the new data provided in SI. Although I understand the communication format does not allow to extend too much the discussion, I am sure it is essential for the reader, who will not have access to the rebuttal letter.

Answer 4. 1) As suggested, the reason why higher temperature was not used for the pulsed chromatography experiment has been appended to line 204-206 with the relevant references.

2) Thanks for the very constructive comment. Now the color of different lines in pulsed chromatography has been harmonized for figures in the Supplementary Information and in the main article. Meanwhile, discussion of the other 2 zeolites pulsed chromatography (now in Supplementary Information Figure 13 has been included in line 223-227 to explain the deficiency of HZSM-5_NK and HZSM-5_C2 for shape selective process.

Question 5. Figure S8: the link between the XPS depth profile (time) to depth in nm should be shown as it is highly relevant.

Answer 5: Thanks for the comment, the link between sputter time and depth has been included in Figure 2 captions, line 323-324 "(based on Ar ion sputtering over Ta_2O_5 film, of which the thickness was reduced by 0.17 nm every second under the same sputtering conditions)."

Reviewer #3

The group has added significant amount of work to address my concern from the previous review. In particular, adding the synthesis method is crucial for the work. Apparently, adding n-butylamine in the synthesis solution has some effects on the morphology and the crystal structure of ZSM-5 catalyst. I hope the group can study the effects of n-Butylamine on the morphology in future and address the intriguing structure-property relationship and how to control it using additional organic molecules.

Answer: Thanks for the comment. As suggested, we would carry out further research on the control of morphology and Al zoning with different organic molecules for even better zeolite performance in the future.
